# Biglycan fragmentation in pathologies associated with extracellular matrix remodeling by matrix metalloproteinases

**DOI:** 10.1186/1755-1536-6-9

**Published:** 2013-05-01

**Authors:** Federica Genovese, Natasha Barascuk, Lise Larsen, Martin Røssel Larsen, Arkadiusz Nawrocki, Yili Li, Qinlong Zheng, Jianxia Wang, Sanne Skovgård Veidal, Diana Julie Leeming, Morten Asser Karsdal

**Affiliations:** 1 Nordic Bioscience A/S, Herlev Hovedgade 207, Herlev, DK-2730, Denmark; 2 Department of Biochemistry and Genetics, Odense University Hospital, Sdr. Boulevard 29, Odense C, 5000, Denmark; 3 Institute for Biochemistry and Molecular Biology, University of Southern Denmark, Campusvej 55, Odense, DK-5230, Denmark; 4 Nordic Bioscience Beijing, Life park road 29, Zhongguancun Life Science Park, Beijing, Changoing district, 102206, China

**Keywords:** Biglycan, Biochemical markers, Extracellular matrix remodeling, Matrix-metalloproteinase, Neo-epitopes

## Abstract

**Background:**

The proteoglycan biglycan (BGN) is involved in collagen fibril assembly and its fragmentation is likely to be associated with collagen turnover during the pathogenesis of diseases which involve dysregulated extracellular matrix remodeling (ECMR), such as rheumatoid arthritis (RA) and liver fibrosis. The scope of the present study was to develop a novel enzyme-linked immunosorbent assay (ELISA) for the measurement of a MMP-9 and MMP-12-generated biglycan neo-epitope and to test its biological validity in a rat model of RA and in two rat models of liver fibrosis, chosen as models of ECMR.

**Results:**

Biglycan was cleaved *in vitro* by MMP-9 and -12 and the 344^′^YWEVQPATFR^′^353 peptide (BGM) was chosen as a potential neo-epitope. A technically sound competitive ELISA for the measurement of BGM was generated and the assay was validated in a bovine cartilage explant culture (BEX), in a collagen induced model of rheumatoid arthritis (CIA) and in two different rat models of liver fibrosis: the carbon tetrachloride (CCL4)-induced fibrosis model, and the bile duct ligation (BDL) model. Significant elevation in serum BGM was found in CIA rats compared to controls, in rats treated with CCL4 for 16 weeks and 20 weeks compared to the control groups as well as in all groups of rats subject to BDL compared with sham operated groups. Furthermore, there was a significant correlation of serum BGM levels with the extent of liver fibrosis determined by the Sirius red staining of liver sections in the CCL4 model.

**Conclusion:**

We demonstrated that the specific tissue remodeling product of MMPs-degraded biglycan, namely the neo-epitope BGM, is correlated with pathological ECMR. This assay represents both a novel marker of ECM turnover and a potential new tool to elucidate biglycan role during the pathological processes associated with ECMR.

## Background

Tightly controlled extracellular matrix (ECM) remodeling is essential for development, wound healing and normal organ homeostasis. However, sustained dysregulation of this remodeling, leading to excessive matrix deposition, can contribute to the onset of life-threatening pathological conditions [1]. The ECM proteins are key players in tissue failure and can become the driving force of the pathogenesis of fibrotic diseases, tumor progression and metastasis [2].

Biglycan (BGN) is a secreted proteoglycan that belongs to the family of small leucine-rich proteoglycans (SLRPs), consisting of a core protein and one or two chondroitin sulfate/dermatan chain(s) bound covalently through a tetrasaccharide bridge to a serine residue. Together with decorin, fibromodulin and lumican, biglycan is a key regulator of lateral assembly of collagen fibers. Biglycan has been shown to specifically interact with type VI collagen by binding the N-terminal region of the triple helix [3]. Deficiency of one or more SLRPs, as well as targeted disruption of the biglycan gene, leads to abnormal collagen fiber diameters and disturbed lateral association of fibers [4]. Biglycan is thought to also have a role in fibrogenesis and in the assembly of elastin fibers [5]. The biglycan core protein contains leucine-rich repeats that facilitate protein-protein interactions: this proteoglycan is in fact able to bind to the membrane-bound proteoglycan dystroglycan and to a wide variety of proteins, being involved in, for instance, cell signal transduction during cell growth and differentiation and in regulating cytokine activity due to its capacity to bind TGF-β and TNF-α [4]. TGF-β1 has been identified as the most pro-fibrotic cytokine, being responsible, for instance, for hepatic stellate cell trans-differentiation into myofibroblast in the first stages of liver fibrosis [6]. By binding to TGF-β1, biglycan is able to inhibit its bioactivity *in vitro*[7]. Moreover it has been demonstrated that the activity of TGF-β1 is strictly related to the presence of biglycan also *in vivo*, as biglycan-deficient mice have shown elevated levels of both total and bioactive TGF-β1 in plasma [8].

Endopeptidases like matrix metalloproteinases (MMPs) play a key role in the degradation of extracellular macromolecules such as collagens and proteoglycans. In the fibrous tissue many MMPs, including MMP-9 and MMP-12, are highly regulated and are responsible for the excessive proteolytic activity [9-11]. The fragmentation of ECM proteins by specific proteases like MMPs, generates small peptides, the so-called neo-epitopes, which may be used as biochemical markers.

The aim of the present study was to identify a pathological neo-epitope originated by MMP-9 and MMP-12-mediated biglycan degradation that potentially is a serological marker for pathological extracellular matrix remodeling (ECMR). Animal models of liver fibrosis were chosen to investigate the relation between this novel biglycan marker and ECMR in fibrosis-related diseases. Furthermore the levels of MMP-degraded biglycan were assessed in an *ex vivo* cartilage explant model, as well as in a rat model of collagen-induced arthritis to test the biological validity of the assay.

## Results

### Selection of neo-epitope by mass spectrometry

**Figure 1:**
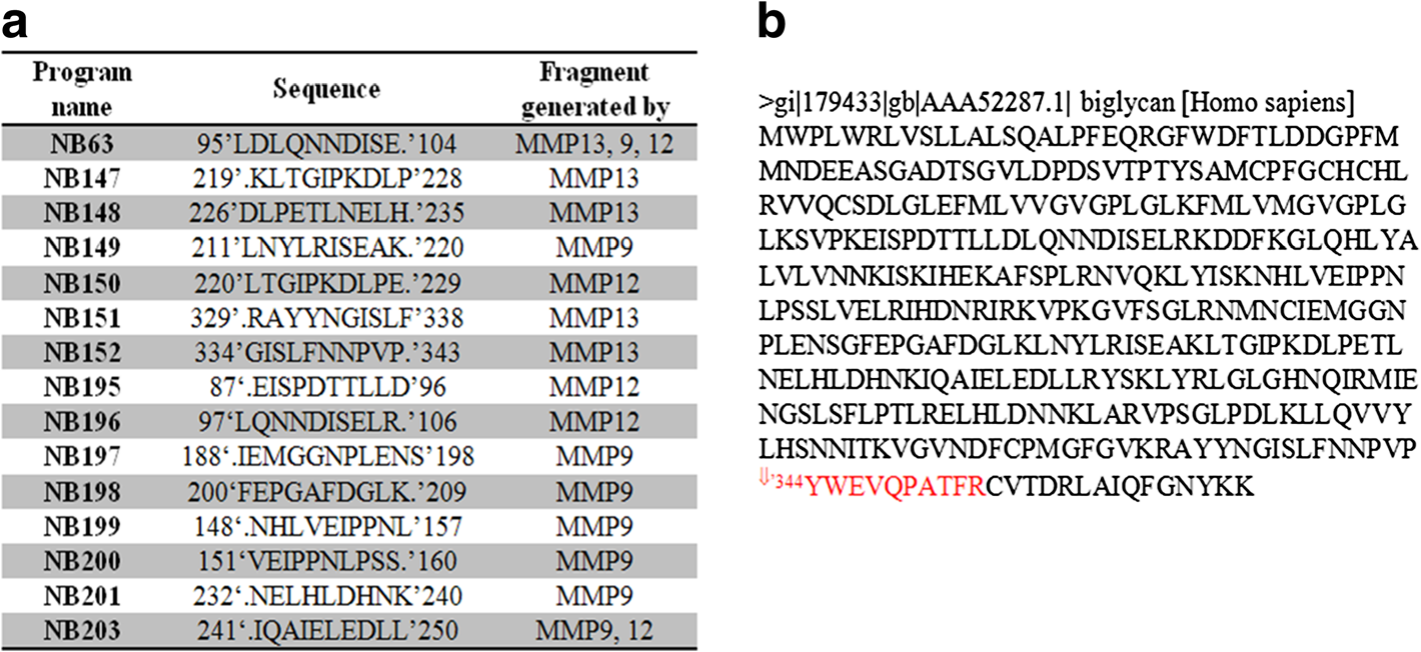
**Cleavage originated peptides and selected BGM.** (**a**) Selection of peptides generated from MMP-9 and -12 cleavages over time. (**b**) Position of the selected peptide BGM in the biglycan sequence.

**Figure 2:**
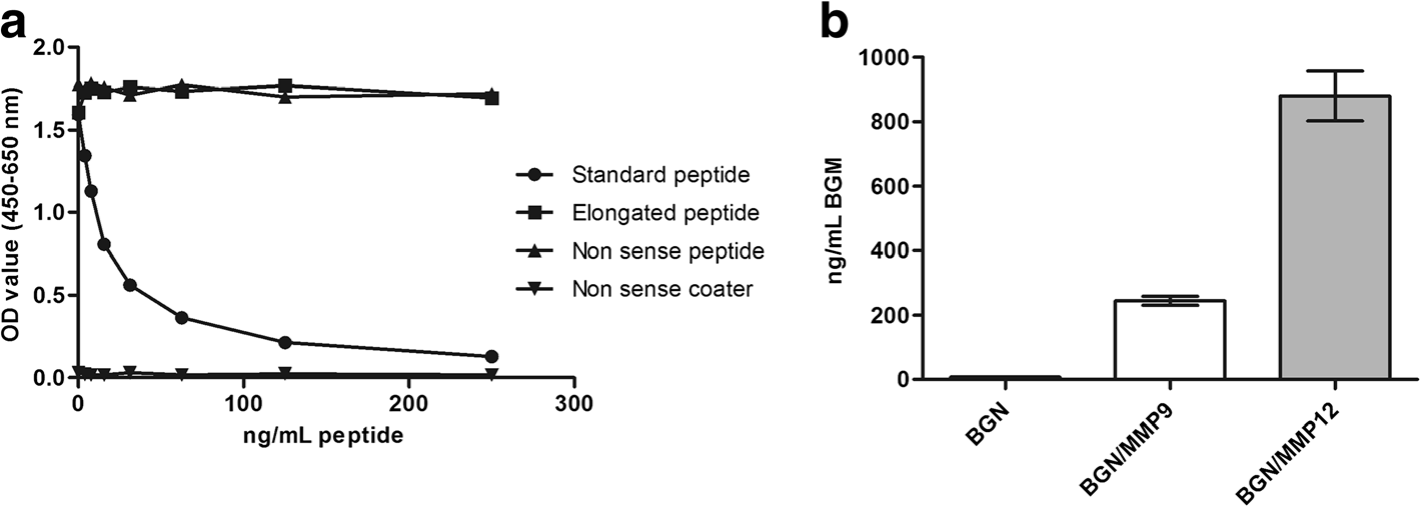
**Technical performance of BGM ELISA.** (**a**) Standard peptide, elongated peptide and non-sense peptide and coater in the BGM competitive ELISA setting (**b**) BGM measurement for *in vitro* MMP-9 and MMP-12-degraded biglycan (BGN/MMP9, BGN/MMP12) and for intact biglycan (BGN).

Purified bovine biglycan was cleaved with a variety of MMPs including MMP-9 and -12, and 120 unique biglycan peptides were identified in the cleaved material. Some of the peptides were generated by both proteases, while others were unique for each protease. The digestion of biglycan over time revealed a time-dependant peptide generation, with some peptides being generated within the first few hours and others after two or three days (data not shown). The length of protease-generated peptides of biglycan was between 10 and 50 amino acids. All peptides were tested for homology and cross-reactivity to other human proteins and across species. Antibodies were generated against sixteen neo-epitope sequences (the non-selected fifteen sequences are shown in Figure 1a), and based on the reactivity against the selection peptide, the specificity for the cleaved biglycan, and the reactivity against native material (human and rat serum), one of the antibodies recognizing one of the peptides identified by LC-MS/MS was selected for assay development. The neo-epitope ^⇓344^YWEVQPATFR^353^ (BGM) (Figure 1b) was generated by MMP-9 and -12, MMP-12 generating the largest quantities of this peptide (Figure 2b). Furthermore, BGM is one of the peptides generated during the early phases of *in vitro* digestion, whereas the largest quantity of the peptide released is seen after 72 hours. The BGM peptide was shown to be unique to biglycan with 100% homology across different species (*Homo sapiens, Mus musculus, Rattus norvegicus, Canis lupus familiaris, Equus caballus, Bos Taurus, Ovis aries*).

### Selection of monoclonal antibody for assay development

From the mouse immunized with the selected sequence, YWEVQPATFR-GGC-OVA, the clone NB202-7-9D6 was selected for assay development due to the highest and most consistent reactivity to both human serum and to biglycan degraded *in vitro* by MMP-9 and -12. The BGM neo-epitope is located only 25 amino acids from the C-terminus, just outside the leucine-rich-repeat area, therefore the peptides recognized by the BGM antibody are expected to be from 6 to 25 amino acids long (Figure 1b).

### Technical performance of the BGM assay

The competitive ELISA assay BGM was developed using the NB202-7-9D6 clone and tested for reactivity to the BGM fragment ^⇓^YWEVQPATFR. Neither the elongated peptide (**P**YWEVQPATFR) nor the non-sense peptide (NNQIDHIDEK) were able to displace the signal, indicating the antibody-antigen reaction was specific to the neo-epitope of the selected biglycan cleavage product. Moreover, no reactivity was shown using a non-sense coater (Biotin-K-NNQIDHIDEK) (Figure 2a). Native, uncleaved biglycan was also incapable of displacing the signal, while only MMPs-cleaved biglycan peptides could inhibit the signal in the assay. Different proteases had different efficiency in cleaving biglycan and generating BGM (Figure 2b). The assay performance is summarized in Table 1. The calculated lower detection limit (LDL) was 1.54 ng/mL. The intra-assay variability was 10% and the inter-assay variability was on average 15%.

**Table 1:** Technical evaluation of the BGM competitive ELISA

**Technical validation step**	**BGM ELISA**
Target	MMP-9 and -12 degradation of biglycan
Lower limit of detection (LDL)	1.54 ng/mL
Dilution recovery of human serum	100% (1:2), 103% (1:4)
Dilution recovery of human plasma	97% (1:2), 99% (1:4)
Dilution recovery of rat serum	107% (1:2), 102% (1:4)
Dilution recovery of rat plasma	89% (1:2)
Dilution recovery of mouse serum	105% (1:2)
Dilution recovery of mouse plasma	97% (1:2)
Intra-assay variation	10%
Inter-assay variation	15%

Percentage dilution recovery and percentage intra/inter variation are indicated as mean values of different samples diluted 1:2 and 1:4 and as mean variation between ten individual determinations of human samples, respectively.

### BGM is produced by bovine cartilage explants *ex vivo*

**Figure 3:**
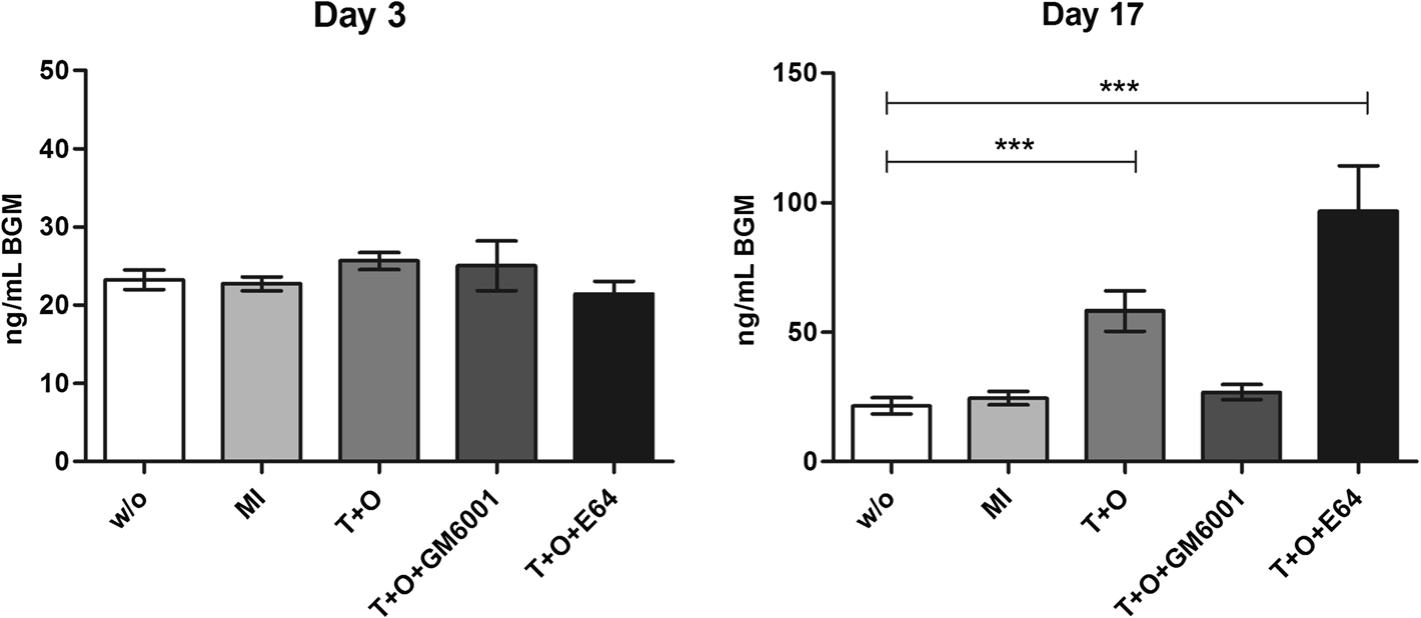
**BGM release from *ex vivo *cultures of bovine explants (BEX), measured at day 3 and 17 of culturing on five replicates.** w/o = cultures without any stimulation; MI = metabolically inactive cultures by the treatment with liquid nitrogen; T+O = catabolic cytokine stimulation with TNF-α (20 ng/mL) and oncostatin (10 ng/mL); T+O+GM6001 = catabolic cytokines TNF-α (20 ng/mL) and oncostatin (10 ng/mL) supplemented with selective MMP inhibitor GM6001; T+O+E64 = catabolic cytokines TNF-α (20 ng/mL) and oncostatin (10 ng/mL) supplemented with selective cysteine protease inhibitor E64. (*** *P* < 0.001).

To investigate the generation of this unique fragment, we performed an *ex vivo* experiment on bovine cartilage explants (BEX) cultured for 17 days in the presence of TNF-α and oncostatin (T+O) or in four other solutions. The addition of catabolic supplements has previously been shown to potently induce time-dependent cartilage degradation by aggrecanases and MMPs [5]. At early time points (three and eight days of culture) no difference in the release of BGM was seen between any of the five culture groups. At the end of culturing period (day 17) a more than two-fold increase in peptide release was observed in the T+O culture group compared to non-stimulated cultures (Figure 3). The selective MMP inhibitor, GM6001, added to the TNF-α and oncostatin culture, abrogated the increased levels of BGM, demonstrating a MMP-dependant release of the neo-epitope. The addition of T+O in presence of the cysteine protease inhibitor E64 significantly (*P* < 0.005) augmented the release of the BGM, as compared to T+O alone.

### CIA model

**Figure 4:**
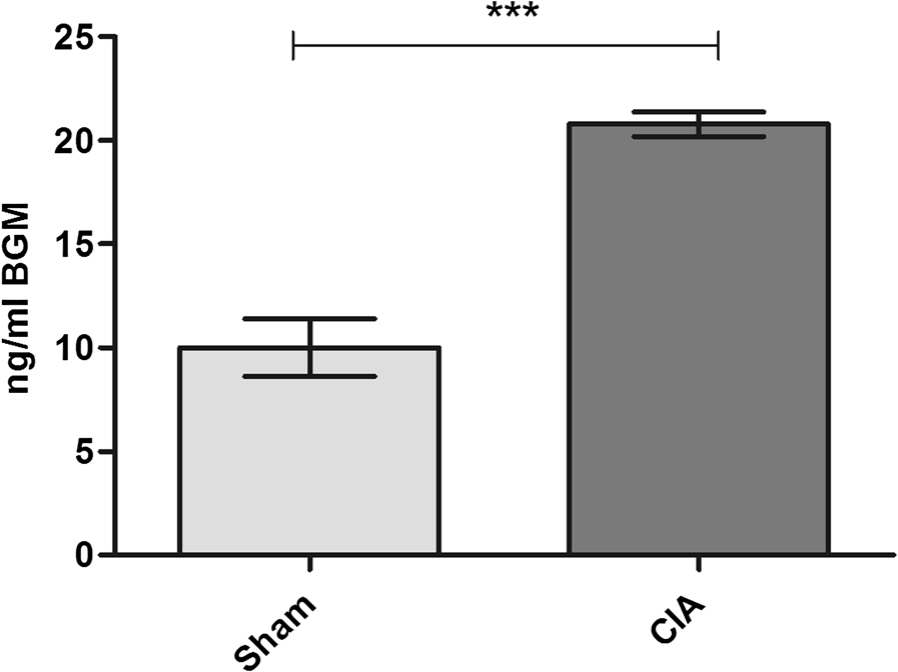
**Mean circulating serum BGM levels in a collagen-induced arthritis (CIA) rat model of rheumatoid arthritis measured at day 22.** Data are shown as mean ± standard error of the mean (SEM) (*** *P* < 0.001).

Serum BGM was investigated at day 22 in a CIA rat model of RA, following previous results that showed high levels of collagen degradation at this time point [12]. Results are presented in Figure 4: serum BGM levels are significantly more elevated in CIA animals compared to controls (sham: 10 ng/mL, CIA: 20 ng/mL, *P* < 0.0001).

### Sirius red staining of livers in rat models of liver fibrosis

**Figure 5:**
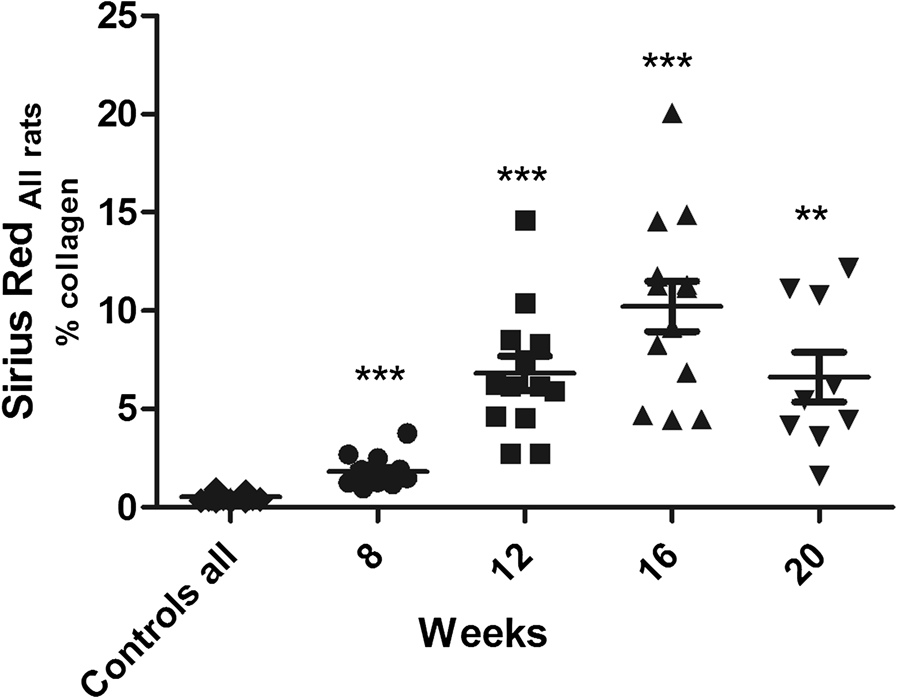
**Absolute values of Sirius red quantification, indicated as % collagen in CCL4-treated rats determined at 8, 12, 16, and 20 weeks of treatment.** (CCL4-treated animals: n = 13; controls: n = 7 for each group). The bars indicate mean ± standard error of the mean (SEM) (** *P* < 0.01, *** *P* < 0.001).

Sirius red staining of livers of CCL4 rats was performed for all animal groups, and the results are presented in Figure 5. The total amount of collagen increases after 12 weeks of CCL4 treatment, peaks at 16 weeks of treatment, and seems to regress to 12-week levels at 20 weeks of treatment.

### BGM for detection of liver fibrosis in rat CCL4 and BDL model

#### CCL4 model

**Figure 6:**
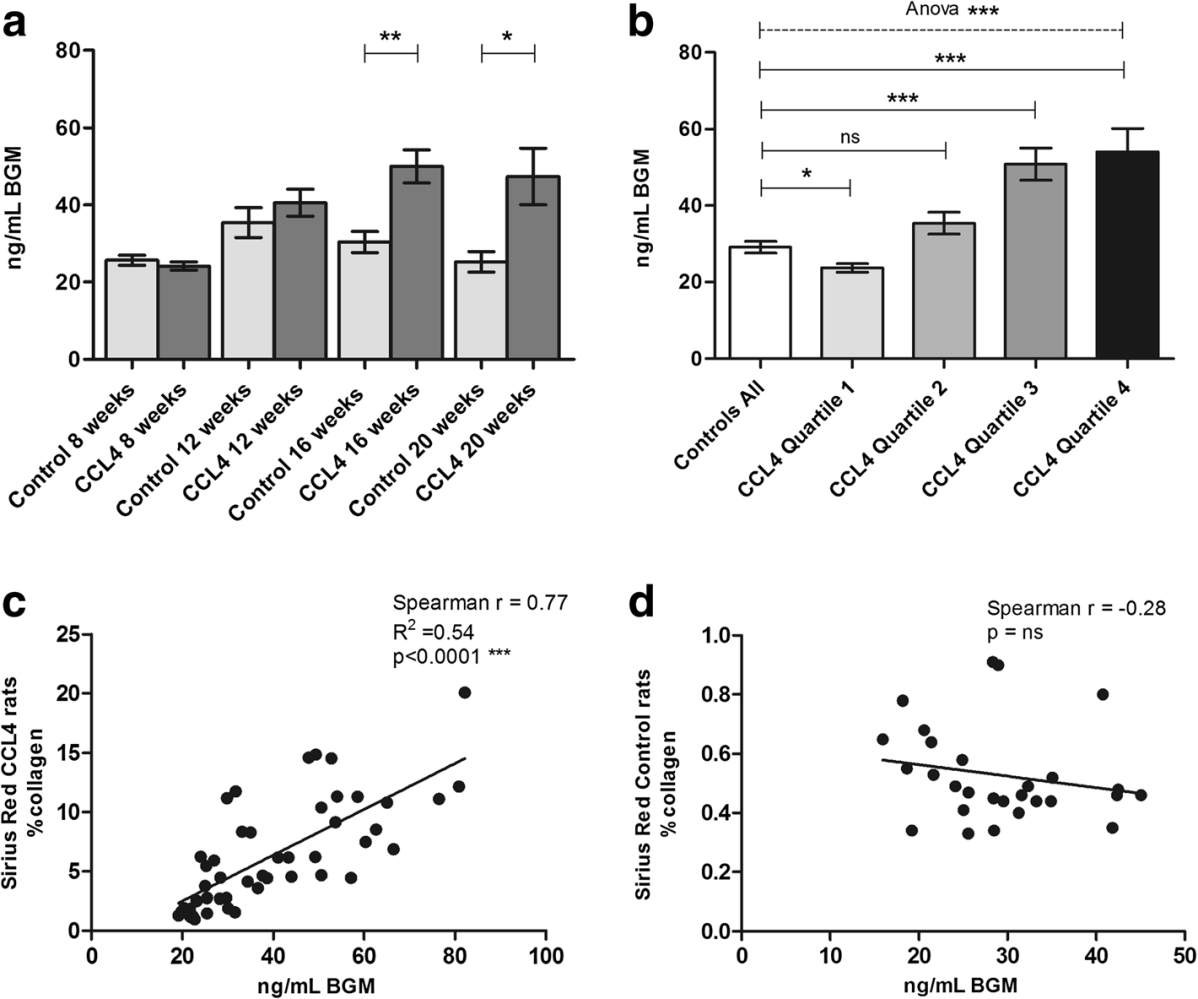
**BGM in CCL4 rats.** (**a**) Mean circulating serum BGM levels in carbon tetrachloride (CCL4)-induced rat model of liver fibrosis measured at 8, 12, 16, and 20 weeks (CCL4_-_treated animals: n = 13; controls: n = 7 for each group). (**b**) Serum BGM levels in all control rats and all CCL4 rats stratified in quartiles according to total collagen content in the liver determined by Sirius red; (ns = non-significant, * *P* < 0.05, ** *P* < 0.01, *** *P* < 0.001). Correlation of circulating serum BGM and histological Sirius red quantification determining the extent of liver fibrosis in (**c**) CCL4-treated rats and (**d**) controls.

After eight weeks, no significant difference was seen in the serum BGM in the groups of 52 male Wistar rats treated bi-weekly with inhalable CCL4 and phenobarbital in drinking water, and the 28 control rats receiving phenobarbital only (Figure 6a). By 12, 16 and 20 weeks, there was an increase in serum BGM levels in CCL4-treated rats, and this increase was found to be significant compared with control rats at 16 weeks (controls: 30 ng/mL, CCL4: 50 ng/mL, *P* = 0.0013) and 20 weeks (controls: 25 ng/mL, CCL4: 47 ng/mL, *P* = 0.019). We found no significant difference in serum levels of control rats during the study period. Correlations of the levels of serum BGM with the percentage of fibrotic tissue determined by Sirius red, indicating the extent of liver fibrosis, are presented in Figure 6c and 6d. As illustrated, we found a significant correlation (r = 0.77, *P* < 0.0001) between levels of serum BGM of CCL4 animals and the extent of their fibrosis. No significant correlation was found in control animals (r = -0.28, *P* = ns). By further data analysis all animals were divided into quartiles according to the extent of fibrosis in the tissue as determined by Sirius red staining, and stratified according to the level of serum BGM (Figure 6b). These data analysis showed that the animals with the highest levels of serum BGM showed the most extensive fibrosis.

#### BDL model

**Figure 7:**
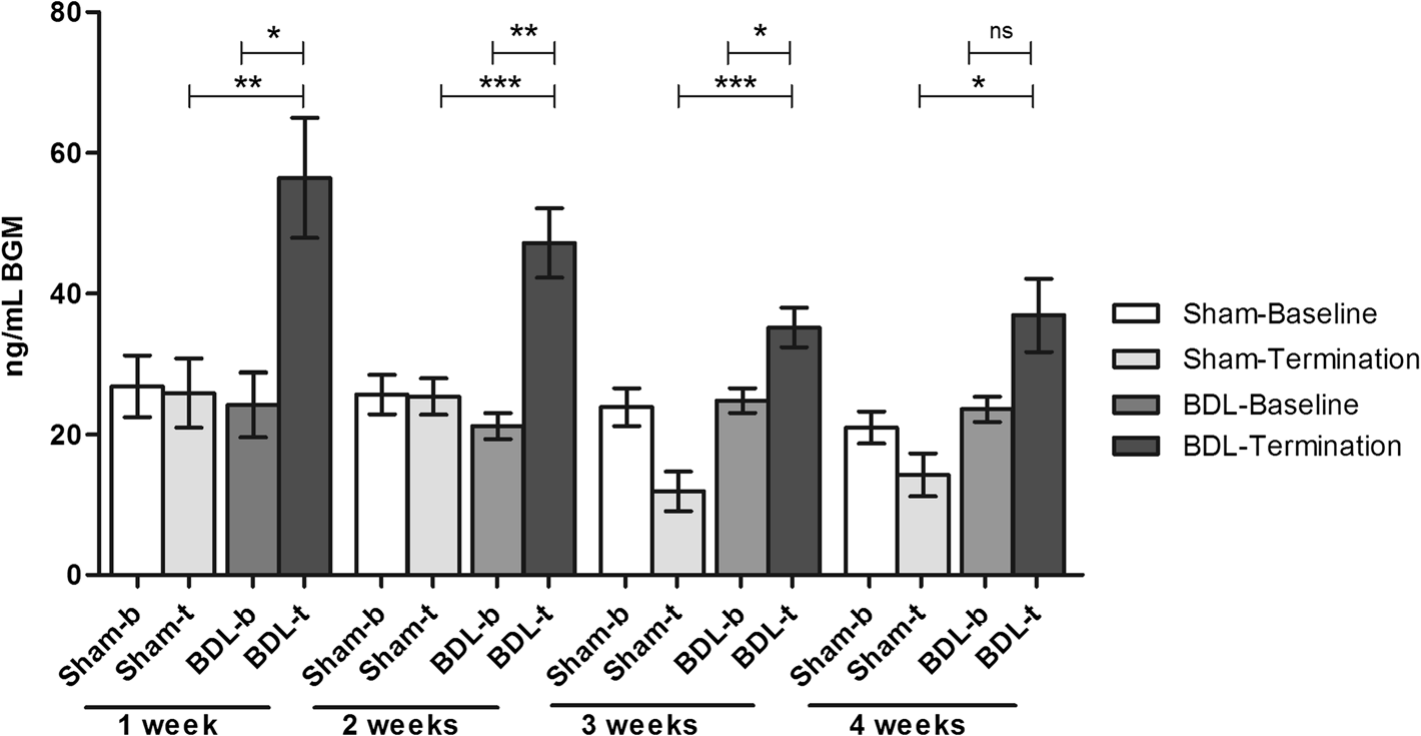
**Mean circulating serum BGM levels in BDL and sham operated rats at baseline and at termination at weeks 1, 2, 3, 4.** Data are shown as mean ± standard error of the mean (SEM) (* *P* < 0.05, ** *P* < 0.01, *** *P* < 0.001).

Serum BGM levels increased significantly in all BDL groups compared with sham groups (group 1: sham 27 ng/mL, BDL 56 ng/mL, *P* < 0.01; group 2: sham 25 ng/mL, BDL 47 ng/mL, *P* < 0.001; group 3: sham 12 ng/mL, BDL 35 ng/mL, *P* < 0.001; group 4: sham 14 ng/mL, BDL 37 ng/mL, *P* < 0.05) (Figure 7). The serum BGM levels in BDL animals were significantly elevated at termination compared to baseline at all time points except at week 4 (week 1 baseline: 24 ng/mL, termination: 56 ng/mL, *P* < 0.01, week 2 baseline: 21 ng/mL, termination: 47 ng/mL, *P* < 0.001, week 3 baseline: 24 ng/mL, termination: 35 ng/mL, *P* < 0,025, week 4 baseline: 23 ng/mL, termination: 37 ng/mL, *P* = 0,07). An increased trend was observed in the marker levels in the early phases of fibrosis that decreased over time from week 2 to weeks 3 and 4, but this was not statistically significant.

## Discussion

MMP degradation of ECM components generates specific cleavage fragments, called neo-epitopes. The combination of a specific protease and a specific ECM protein component, namely protein fingerprint, may provide a unique combination that can be relevant for a certain pathology and be ascribed to a specific tissue [13]. This class of biomarkers has proven successful in studies on osteoarthritis and osteoporosis [14], liver [15,16] and skin fibrosis [17]. Collagen protein fingerprints have already been used to generate novel neo-epitope markers of ECM remodeling [18-20], and considering the role of biglycan as collagen assembly regulator in many tissues, we hypothesized that biglycan is also remodeled during the same pathological processes that lead to dysregulated ECM turnover. To validate this hypothesis, we developed an immunological assay detecting a neo-epitope of biglycan generated by MMP-9 and MMP-12 cleavage (BGM) in serum, and measured the levels of this marker in one rat model of RA and in two rat models of liver fibrosis, chosen as model pathologies involving disrupted ECM turnover.

Biglycan is abundant in the ECM of many tissues and it has been shown to be up-regulated, together with MMPs, in fibrotic livers [21] and in related diseases such as lung, heart and kidney fibrosis [22-25]. Despite its functions in collagen assembly and as a signal molecule implicated in cell adhesion, migration, differentiation and apoptosis, have been demonstrated *in vitro*, biglycan biological roles *in vivo* have not yet been fully understood [26].

The ECM is a very complex environment, in which different proteins such as collagens, proteoglycans and proteases act together to maintain the equilibrium between ECM degradation and formation. Many proteases, including MMPs, are involved in the intricate mechanism of fibrogenesis *in vivo*, each of them contributing to the proteolysis of different ECM proteins. The *in vivo* interplay that occurs between different types of proteases can be successfully simulated by *ex vivo* models [5]. In this study, we performed an *ex vivo* experiment on bovine cartilage explant cultures using the developed assay to measure the levels of BGM generated in the cultures. Cartilage degradation in these cultures is known to follow a time-dependent path, in which firstly aggrecanase (ADAMTS), and later MMP activity is responsible for the catabolic destruction of the cultures [27,28]. Bovine cultures stimulated with T+O released the highest quantities of the BGM neo-epitope during the MMP induction period, but this release was completely abrogated by the addition of the specific MMP inhibitor, GM6001, demonstrating that the generation of BGM is MMP-dependent. Interestingly, in the presence of T+O and a cysteine protease inhibitor, we found an increase rather than a decrease in BGM levels. This observation suggests compensatory or feedback mechanisms are part of a complex interplay between the proteases *in vivo*. We have previously demonstrated that there is an increase in MMP-9 activity in the absence of the cysteine protease Cathepsin K (CatK) in CatK null-mutation mice [12]. This suggests that caution should be directed to therapies directed at the inhibition of protease activity due to the potential for resulting compensatory mechanisms.

Following the results in the cartilage model, we measured the serum levels of BGM in an animal model of RA, an autoimmune disease which causes chronic inflammation leading to ECMR in the synovial joints. The formation and degradation profile of different types of collagen has previously been studied in the CIA model, showing an increase in collagen degradation neo-epitope levels in serum with the progression of the disease [29]. Considering the close association of biglycan with collagen, we evaluated the potential of BGM as a marker for ECMR in this model. The results show a good separation between healthy and diseased animals in the levels of BGM in the serum, suggesting that this proteoglycan could also be important in the development of the pathology. To further confirm the relationship of BGM with ECMR, two animal models of liver fibrosis, the CCL4-treated rats and the BDL rats, were investigated to examine the levels of the biglycan neo-epitope as well as its potential relation to fibrosis. Serum BGM levels were significantly correlated with the extent of liver fibrosis judged by histological Sirius red quantification in CCL4-treated rats. No correlation was observed between Sirius red determination of liver fibrosis extent and levels of BGM in control rats. In the CCL4 model of liver fibrosis, serum BGM was elevated after 16 and 20 weeks of treatment compared with controls and these data are in agreement with the literature stating that biglycan is highly deposited in sites affected by fibrosis [21], where MMP levels are elevated and unbalanced during fibrogenesis [30]. This pattern is very similar to that of other ECM degradation markers in this model, as shown by means of Z-score plots in the paper by Leeming *et al.*[31]. The findings obtained in the CCL4 model were confirmed in the BDL model, where the levels of serum BGM were elevated to a larger extent in BDL rats compared to sham-operated rats at all time points. However this model shows a different expression pattern compared to the CCL4 model: after an initial peak of serum BGM in BDL operated animals one week after the treatment, there is a non-statistically significant trend of decreasing marker levels at week 4. These results however, are not surprising, as the two rodent models represent different types of human fibrosis. Bile duct ligation (BDL) rats are models of chronic liver inflammation similar to what has been observed in human cholestatic liver disease. Carbon tetrachloride (CCL4) treatment on the other hand causes acute liver damage, providing a model resembling the human condition of alcoholic steatohepatitis with the consequent fibrosis and cirrhosis [30,32]. Our working hypothesis is that BGM is a marker of fibrosis activity, able to reflect the levels of ECMR activity and the overall remodeling that occurs in an organ. The remodeling outcome can, in turn, depend on the organ and the insult, which may vary according to the nature of the treatment and of the organ system that is affected.

## Conclusions

In this work, we have developed the first assay to measure a pathologically relevant fragment of biglycan in biological fluids, using a specific monoclonal antibody for the detection of BGM, a biglycan fragment derived from MMP-9 and MMP-12 activity, in human, rat and mouse serum. We have demonstrated that this serum marker is elevated in a rat model of RA and in two rat models of liver fibrosis and it is highly correlated with the extent of fibrosis, suggesting serum BGM is a relevant biomarker for ECMR. This assay enables the analysis of biglycan degradation in both animal studies and potentially in clinical settings. The use of this assay in clinical studies of diseases involving ECM remodeling might be useful to elucidate the role that biglycan exerts not only in the complex tissue environment during the pathological processes that cause RA and fibrosis, but also in other tissues where biglycan is expressed, such as muscle, bone, teeth and tendon.

## Methods

### Materials

All chemicals, enzymes and cell culture reagents were purchased from Sigma-Aldrich (Copenhagen, Denmark), or VWR (Herlev, Denmark), if not otherwise stated. The ELISA plates, pre-coated with streptavidin (Nunc clear 96-well plates), were purchased from Roche Diagnostics (Mannheim, Germany). Immunogens, standard and coating peptides were purchased from the Chinese Peptide Company (Beijing, China) and from American Peptide (Sunny Valley, CA, USA).

### *In vitro* peptide generation

The BGM neo-epitope was identified by *in vitro* degradation of bovine articular cartilage purified biglycan (Sigma-Aldrich, Copenhagen, Denmark) by MMP-9 and -12. The purified biglycan had been filtered to remove proteins below 10,000 kDa (Microcon Ultracel YM-10, catalog number. 42407, Millipore, Billerica, MA, USA) and had not been de-glycosylated prior to MMP digestion. The buffer used for the MMP cleavage of biglycan (1 mg/mL) consisted of 100 mM Tris–HCl, 100 mM NaCl, 10 mM CaCl_2_ and 2 mM ZnAc, at pH 8.0. The cleavage fragments were obtained after 72 hours of incubation with each protease. As a control biglycan (1 mg/mL) was incubated with MMP-buffer. The cleavages were stopped by 5 mM EDTA and verified by SDS-PAGE.

### Peptide identification and antibody generation

After the *in vitro* cleavage, peptides of biglycan were identified using liquid chromatography coupled to electrospray ionization (ESI) tandem mass spectrometry (LC-MS/MS) as previously described [15]. To identify peptides, MS and MS/MS data were searched against a biglycan (FASTA) protein database using the Mascot 2.2 (Matrix Science, Boston, MA, USA) software with ESI-QUAD-TOF settings and carbamidomethyl (C), oxidation of methionine (M), oxidation of lysine (K) and oxidation of proline (P) as variable modifications. The first six amino acids of each free end of the protease-generated peptide sequences identified by MS were regarded as a neo-epitope generated by the specific protease.

All MMP-9 and -12 generated neo-epitopes were analyzed for distance to other cleavage sites and then blasted for protein and species homology using the NPS@: network protein sequence analysis [33]. Among all the different neo-epitopes, the sequence ^344^YWEVQPATFR^353^ was chosen according to the mentioned criteria. A monoclonal antibody targeted against the N-terminal part of the selected peptide was generated as previously described [20].

### BGM ELISA development

A competitive ELISA for the biglycan selected neo-epitope BGM was developed as follows: a 96-well streptavidin-coated plate was coated with 2.5 ng/mL biotinylated synthetic peptide YWEVQPATFR-K-Biotin dissolved in PBS buffer (2 mM KH_2_PO_4_, 9 mM Na_2_HPO_4_, 2H_2_O, 3 mM KCl, 137 mM NaCl, pH 7.4) and incubated for 30 min at 20°C by constant shaking at 300 rpm. 20 μL of peptide calibrator prepared by two-fold pre-dilution of the standard peptide (YWEVQPATFR) starting from 250 ng/mL or sample dissolved in assay buffer (2 mM KH_2_PO_4_, 9 mM Na_2_HPO_4_, 2H_2_O, 3 mM KCl, 34 mM NaCl, 5% Osteocalcin EIA Puf-Liq (Roche Diagnostics Deutschland GmbH) pH 7.4) were added to appropriate wells, followed by 100 μL of 40 ng/mL peroxidase-labeled NB202-7 9D6 antibody and incubated for one hour at 20°C by constant shaking at 300 rpm. Finally, 100 μL of tetramethylbenzidine (TMB) (catalog number 438OH; Kem-En-Tec, Copenhagen, Denmark) were added, and the plate was incubated for 15 minutes at 20°C in the dark and shaken at 300 rpm. After each incubation step, the plate was washed five times in washing buffer (20 mM Tris, 50 mM NaCl, pH 7.2). The TMB reaction was stopped by adding 100 μL of stopping solution (1% HCl) and the colorimetric reaction was measured at 450 nm with reference at 650 nm on a standard laboratory plate reader. Data were acquired with the SoftMax Pro v5.0 (Molecular devices, LLC, Sunnyvale, CA, USA) program.

### Technical evaluation of BGM assay

Technical assay validation was performed according to international guidelines of assay development. Briefly, linearity was calculated as a low, medium or high percentage of recovery of the 100% sample from two-fold dilutions of quality control (QC) human serum and from rat serum. The lower limit of detection (LDL) was determined from 21 zero samples (buffer only) and calculated as the mean + 3 x standard deviation. Percentage dilution recovery was calculated as the mean of five human serum and five human plasma samples, four rat serum and four rat plasma and three mouse serum and three mouse plasma diluted 1:2 and 1:4. Inter- and intra-assay variations were calculated as the mean variation between ten individual determinations of eight QC samples (human serum) with each run consisting of two replicas of double determinations of the samples.

### ELISA characterization

The developed BGM ELISA was evaluated using 20 μL of the samples: intact biglycan, biglycan cleaved with MMP-9, biglycan cleaved with MMP-12, the standard BGM peptide YWEVQPATFR and the BGM peptide elongated at the N-terminal end with one amino acid (PYWEVQPATFR). Specificity was tested using a non-sense peptide NNQIDHIDEK and a non-sense coater Biotin-K- NNQIDHIDEK.

### Bovine cartilage explant cultures

Bovine cartilage explants (BEX) were harvested by dissecting the outermost layer of articular cartilage from bovine knee joints, as previously described [34]. The cartilage explants (16 ± 4 mg) were placed in 96-well plates and incubated at 37°C, with 5% CO_2_ and shaken at 50 rpm under serum-free conditions (n = 5 per condition). Each explant was cultured in 200 μl of DMEM for seventeen days, with the medium being changed every three to four days, under one of the following conditions: 1) Without catabolic factors (W/O), 2) Metabolically inactivated by liquid nitrogen, 3) With the catabolic cytokines oncostatin M (10 ng/mL) and TNF-α (20 ng/mL), O+T to stimulate MMP activity; 4) O+T supplemented by the MMP inhibitor GM6001 (10 μM); and 5) O+T supplemented by the cysteine protease inhibitor E64 (50 μM), here used as a negative control, as the selective cathepsin inhibitor should not have an effect on MMP activity. Each condition was replicated five times. The metabolic activity (viability) of the articular explants was quantitatively measured on the last day in culture, using the Alamar Blue assay (TREK Diagnostic Systems, LTD. East Grinstead, West Sussex RH19 1XZ UK) according to the manufacturer’s instructions (data not shown).

### Collagen-induced arthritis (CIA) model

Levels of BGM were measured in a CIA rat model. Complete details of the study have been previously described [29]. The animal experiment protocol was approved by the local animal ethics committee at Nordic Bioscience Beijing. The ethical approval number is NBB-AM-R/2009-01. Briefly, CIA was induced in ten seven-week old female Lewis rats by immunizing with 450 μl 2 mg/mL porcine type II collagen dissolved in 0.05 M acetic acid and emulsified 1:1 in incomplete Freund’s adjuvant on day 0 and 7. Ten Lewis rats, injected only with 0.05 M acetic acid, were used as control. Every day, starting from day 8, rats were examined for visual signs of disease, defined as macroscopic evidence of increase in paw size. The rats were sacrificed on day 26. Serum samples were collected throughout the experiment from overnight fasted animals.

### Rat model of CCL4-induced liver fibrosis

Serum BGM levels were measured in a CCL4 inhalation rat model of liver fibrosis. Complete details of the study have been previously described [35]. The CCL4 study was approved by the Ethical Committee of Animal Experimentation of the University of Barcelona (B-NNP-233/09) and was performed according to the criteria of the Investigation and Ethics Committee of the Hospital Clinic Universitari (Barcelona, Spain). The study included 52 male Wistar rats treated with CCL4 and 28 male Wistar control rats (Charles-River, Saint Aubin les Elseuf, France). Induction of liver fibrosis was performed as previously described [36]. Briefly, CCL4 was administered by inhalation twice weekly and phenobarbital (0.3 g/l) added to the drinking water. Control rats received phenobarbital only. Animals were stratified into groups receiving CCL4 or control treatment for 8, 12, 16 or 20 weeks (n = 13 for CCL4; n = 7 control for each group). Four animals from the CCL4 groups died during the study. Blood was collected at termination and allowed to stand at room temperature for 30 minutes to allow clotting, before centrifugation at 3,000 *g* for 10 minutes. All clot-free liquid was transferred to new tubes and centrifuged again at 3,000 *g* for 10 minutes. Samples were stored at -80°C prior to biomarker assessment. Liver sections (4 μm thick) were stained in 0.1% Sirius red F3B (Sigma-Aldrich, St. Louis, MO, USA) in saturated picric acid (Sigma-Aldrich St. Louis, MO, USA). From each animal analyzed, the amount of fibrosis was expressed as a percentage of the total liver area of 36 fields and the average value is presented. Each field was acquired at 10 x magnification [37].

### Rat model of bile duct ligation (BDL) induced liver fibrosis

Serum BGM levels were measured in a rat model of liver fibrosis induced by bile duct ligation. Complete details of the study have been previously described [18,35]. The BDL experiment was approved by the Experimental Animal Committee of the Danish Ministry of Justice and was performed according to the European Standard for Good Clinical Practice (2008/561-1450).

The study included a total of 81 female Sprague–Dawley rats aged six months. Liver fibrosis was induced in anaesthetized rats by standard BDL in which the bile duct was ligated in two places and dissected between the ligations prior to closing the abdomen. In sham-operated rats, the abdomen was closed without BDL. The rats were divided into four groups: group 1 (10 BDL, 8 sham) was sacrificed after one week, group 2 (12 BDL, 8 sham) sacrificed after two weeks, group 3 (13 BDL, 8 sham) sacrificed after three weeks, and group 4 (14 BDL, 8 sham) sacrificed after four weeks. During the four weeks, 15 of 81 rats, 14 of them BDL operated, were terminated due to excessive weight loss.

### Statistics

The ELISA standard curve was fitted by the four-parameter method:

$$y=\left(A-D\right)/\left(1+{\left(x/C\right)}^{\wedge }B\right)+D,\;\mathrm{where}\;R>0.9$$

Comparison between measurements of biomarkers in culture supernatants and differences between tertiles were assessed by one-way ANOVA with Dunnett’s post-test assuming Gaussian distribution, on accumulated data. Comparison of two subject groups was made using the non-parametric Mann–Whitney test, α = 0.05. The correlations coefficient was calculated using the Spearman’s ρ non-parametric test. GraphPad Prism v.5 (GraphPad Software, Inc. La Jolla, CA 92037 USA) was used for drawing graphs and calculating statistics.

## Abbreviations

BDL: bile duct ligation; BEX: bovine cartilage explant; BGM: biglycan degraded by MMPs; BGN: biglycan; CatK: cathepsin K; CIA: collagen-induced arthritis; CCL4: carbon tetrachloride; DMEM: Dulbecco’s modified eagle’s medium; ECM: extracellular matrix; ECMR: extracellular matrix remodeling; EDTA: ethylenediaminetetraacetic acid; ELISA: enzyme-linked immunosorbent assay; ESI: electrospray ionization; LC-MS/MS: liquid chromatography coupled with tandem mass spectrometry; LDL: lower limit of detection; MI: metabolically inactive; MMP: matrix metalloproteinase; QC: quality control; RA: rheumatoid arthritis; SLRPs: small leucine-rich proteoglycans; TGF-β: transforming growth factor β; TMB: tetramethylbenzidine; TNF-α: tumor necrosis factor α; T+O: TNF-α+oncostatin

## Competing interests

FG, NB, LL, QZ, YL, JW, SSV, DJL and MAK are full time employees at Nordic Bioscience. All other authors have no conflicts of interest.

## Authors’ contribution

FG, NB and SSV have conceived and designed the experiment, FG, NB, LL, YL, QZ, AN and JW have performed the experiments. FG, NB, SSV and AN have analyzed and interpreted the data. FG and NB have drafted and revised the manuscript. MRL, SSV, MAK and DJL have critically revised the manuscript and have given intellectual contribution to the content. All authors have given final approval of the latest version of the manuscript.
